# Health and environmental impacts of shifting to plant-based analogues: a risk-benefit assessment

**DOI:** 10.1007/s00394-025-03749-z

**Published:** 2025-07-05

**Authors:** Catarina Carvalho, Daniela Correia, Sofia Almeida Costa, Rita Pereira, Andreia Oliveira, Elisabete Pinto, Carla Lopes, Duarte Torres

**Affiliations:** 1 EPIUnit– Instituto de Saúde Pública da Universidade do Porto, Rua das Taipas, nº 135 4050-600 Porto, Portugal; 2 Laboratório para a Investigação Integrativa e Translacional em Saúde Populacional (ITR), Porto, Portugal; 3 Faculdade de Ciências da Nutrição e Alimentação, Universidade Do Porto, Porto, Portugal; 4 Departamento de Ciências da Saúde Pública e Forenses e Educação Médica, Faculdade de Medicina, Universidade do Porto, Porto, Portugal; 5 CBQF-Centro de Biotecnologia e Química Fina-Laboratório Associado, Escola Superior de Biotecnologia, Universidade Católica Portuguesa, Porto, Portugal

**Keywords:** Plant-based analogues, Health impact, Environmental impact, Risk–benefit assessment, Ultra-processed foods

## Abstract

**Purpose:**

Plant-based analogues (PBAs) simulate animal-based foods’ attributes and can facilitate adherence to flexitarian and vegetarian dietary patterns, which have been associated with health benefits. However, possibly classifying PBAs as ultra-processed (UPF) and excluding healthy animal-based foods (e.g., fish) can result in unintended health risks. This study aims to quantify the health and environmental impact of replacing animal-based foods with PBAs.

**Methods:**

Using data from the Portuguese National Dietary Survey (n = 3852 adults; 2015–2016), three substitution scenarios of animal-based foods with PBAs were modelled: vegan (replacing all animal-based foods with PBAs), ovolactovegetarian (replacing meat and fish with PBA), and pescatarian (replacing meat with PBA). Varying degrees of substitution (33%, 50%, 67%, 100%) and two classification approaches for PBAs were explored: UPF or non-UPF. The overall health impact was estimated considering several health outcomes (cancer, cardiovascular diseases and metabolic outcomes) combined through Disability-Adjusted Life Years (DALYs). The environmental impact was measured through greenhouse gas emissions and land use.

**Results:**

Environmental benefits were evident, especially for the vegan scenario. Regarding health impact, the 100% substitution of all animal-based foods (vegan scenario) might represent a risk if PBAs are classified as UPF (∆DALY _average_ = 72,109 years). The highest overall benefit was found for 100% substitution of meat only (pescatarian scenario) independently of considering PBAs as UPF or not (UPF: ∆DALY _average_ =  − 40,202 years; non-UPF: ∆DALY _average_ =  − 88,827 years).

**Conclusion:**

PBAs can be considered feasible alternatives to animal-based foods, and the results emphasise meat substitution as a crucial factor for health and environmental benefits.

**Supplementary Information:**

The online version contains supplementary material available at 10.1007/s00394-025-03749-z.

## Introduction

Animal-based foods, especially meat and meat products, are the major contributors to dietary protein intake in European countries [1]. Nevertheless, meat consumption has been associated with a higher risk of several chronic diseases and a higher potential for environmental degradation [2, 3]. Thus, in recent years, the need for a global shift from dietary patterns rich in animal-based foods towards more plant-based ones to achieve health and environmental goals has been advocated [4].

Accompanying these issues, the plant-based food analogues (PBAs) market has grown steadily over the past years. A report from the Smart Protein project has shown a growth of 49% over two years, from 2018 to 2020, for plant-based meat, milk, yoghurt, cheese, ice cream, and fish in Europe [5]. PBAs are foods that mimic their animal-based counterparts’ physicochemical, functional, and sensory attributes [6]. By simulating animal-based foods’ flavour, texture and appearance, PBAs can facilitate the transition towards more sustainable dietary patterns without substantial changes in the established meal consumption patterns [6, 7].

Even though the environmental impact of PBAs is higher than that of other, less processed, plant-based foods, it is still, on average, 50% lower than animal-based foods [8]. Regarding the health impact of PBAs, the evidence linking PBAs to health outcomes is still scarce, with a recent systematic review emphasising the heterogeneity, short duration and limited scope of studies addressing the impact of PBAs on health [9]. Nevertheless, the transition to dietary patterns that replace animal-based foods with PBAs is expected to have some benefits due to decreased levels of meat consumption and increased fibre intake. Still, not all animal-based foods present risks to human health. For instance, fish consumption has been associated with multiple health benefits on cardiovascular health and neurodevelopment [10, 11], and animal-based foods are the primary sources of some nutrients, such as high-biological-value protein, complex B vitamins, vitamin D, calcium, iron, and zinc [12]. Thus, the arbitrary substitution of all animal-based foods is not free of potential risks due to nutrient inadequacy.

Furthermore, according to the definition of ultra-processed foods (UPF) (i.e., formulations of ingredients, mostly of exclusive industrial use, that result from a set of industrial processes [13]), most PBAs would be considered UPF. Given that increased consumption of UPFs has been identified as a risk factor for several non-communicable diseases, overweight, and obesity [14], one could hypothesise that if PBAs are classified as UPF, shifting from animal-based foods to PBAs could entail some health risks that should be ascertained. Nevertheless, most evidence collected on the associations between UPF consumption and health outcomes results from foods that are not PBAs, and there is no clear evidence that the major mechanisms linking UPF to poor health outcomes (i.e., high energy density, hyper-palatability and low satiety potential) apply to PBAs [15]. Consequently, gaps in the evidence remain on whether the potential health effects associated with conventional UPF can be extrapolated to PBAs [15].

This study aims to comprehensively assess the health and environmental impacts of the Portuguese population’s transition from animal-based foods to PBAs by applying a risk–benefit assessment (RBA) methodology. The study explores various alternative scenarios, reflecting different levels of food substitution, including a complete shift to a hypothetical vegan (replacing all animal-based foods with PBAs), ovolactovegetarian (substituting meat and fish with PBAs), and pescatarian (replacing only meat with PBAs) diets. Furthermore, this research also investigates the potential impact on the study outcomes by considering PBAs as a category of UPF.

## Methods

### Study participants and food consumption data

Data from the Portuguese Food, Nutrition and Physical Activity Survey (IAN-AF 2015–2016), a nationwide survey of the non-institutionalised general population of Portugal, was used in this study. The survey involved 5811 subjects aged three months to 84 years who completed two dietary assessments. Multistage sampling, stratified by the seven Statistical Geographic Units of Portugal (Nomenclature of territorial units for statistics—NUTS II), was used to select a representative sample of participants from the Portuguese National Health Registry according to sex and age group (< 1 year, 1–2 years, 3–9 years, 10–17 years, 18–34 years, 35–64 years, 65–74 years, and 75–84 years). A complete description of the IAN-AF 2015–2016 methodological protocol can be found in other publications [16, 17]. For this study, the analyses were restricted to the adult participants (≥ 18 years, n = 3852) of IAN-AF 2015–2016.

The food consumption data were collected according to the harmonised EU Menu guidelines [18]. For the adult participants, trained nutritionists conducted two non-consecutive face-to-face computer-assisted interviews, where a 24-h recall was applied.

During the interview, detailed data on foods, recipes, dietary supplements, and the respective quantification were collected using a validated electronic tool (eAT24) specifically designed for the survey [19]. The FoodEx2 system [20] was used for food and recipe classification and description, while the NOVA classification was applied to categorise foods based on the degree of food processing [13]. The NOVA coding was conducted independently by two researchers. Then, a third researcher verified both coding lists to identify discrepant items. Later, a discussion among all team members was conducted to classify these items by consensus. In case of doubtful classification, the experts decided on the most conservative classification, the one corresponding to the lowest processing level.

### Definition of RBA scenarios—plant-based analogues

A full description of the reference and alternative scenarios pictured for this RBA is presented in Table 1.

**Table 1:** Characterisation of reference and alternative scenarios of the plant-based analogues (PBAs) risk–benefit assessment, using data from the Portuguese Food, Nutrition and Physical Activity Survey (IAN-AF 2015–2016)

Scenario	Description	Proportion (%) of animal-based foods replaced by PBAs
Reference	Current consumption from the IAN-AF 2015–2016	–
Alternative	Substitution of:	
Vegan	All animal-based foods (meat, fish, eggs, dairy, animal fats) by PBAs in different proportions	33%; 50%; 67%; 100%
Ovolactovegetarian	Meat and fish by PBAs, in different proportions Dairy, eggs, and animal fats remain equal to the reference scenario	33%; 50%; 67%; 100%
Pescatarian	Meat by PBAs, in different proportions Fish, dairy, eggs, and animal fats remain equal to the reference scenario	33%; 50%; 67%; 100%

Three alternative substitution scenarios were pictured to evaluate the impact of PBAs: a *Vegan*, an *Ovolactovegetarian,* and a *Pescatarian*. Four versions of each scenario were modelled, reflecting different proportions of substitution (proportions: 33%, 50%, 67%, 100%). The selected proportions were chosen as a practical way to capture a wide range of substitution levels, providing a representative gradient of substitution effects. The substitutions were random, isocaloric, and conducted at the food item level (i.e., specific food items within meals were randomly selected from the consumption data and replaced by a corresponding specific PBA type, varying the amount if needed so that the final energetic value of the meal was constant).

To implement these substitutions, a database of PBAs marketed in Portugal and Europe, including the foods’ nutritional composition, was compiled from the web, namely from supply chain and food industry websites, the European Food Information Resource (EuroFIR), and the Portuguese Food Composition table [21]. The compiled dataset included 384 food items, which were categorised into different PBA types, namely *tofu, seitan*, *specific meat analogues* (including chunks, burgers, nuggets, meatballs, minced, sausages, ham, among others), *specific fish analogues* (including burgers, breaded, other patties, among others)*, fats, vegetable beverages, vegetable yoghurts, vegetable cream, cheese analogues, and egg analogues.* Then, the average nutritional value per type (per 100 g of food) was computed. A substitution matrix was created to ensure that the selected animal-based food from the consumption data would be replaced by a specific analogue (i.e., for example, cheese (animal-based) was only replaced by cheese analogue, ham (animal-based) only replaced by ham analogue, and so on). In these substitutions, plant-based foods such as seitan or tofu, which do not reflect the definition of a specific PBA, were still used for substitution. This decision was grounded in the fact that, in Portugal, these foods are commonly consumed in the same context as specific animal-based foods. For instance, it is common that some traditional Portuguese meat or fish dishes have a plant-based version, typically made with tofu or seitan. For example, “Tofu à Brás” is a typical vegetarian version of the traditional “Bacalhau à Brás”, where tofu is consumed instead of codfish; another example is the substitution of pork meat with seitan in the vegetarian version of the Portuguese Pork and Clams dish.

### Literature review—selection of health outcomes

A background search was conducted to find and review the associations between foods and food components (i.e., macro and micronutrients) expected to vary within alternative scenarios with health outcomes. The search was performed in the PubMed database considering the baseline expression *(((animal_foods) OR (plants_foods) OR (macro OR micronutrients)) AND (health outcomes))), by single reviewers, and* concluded in May 2022. The full expression is presented in Figure S1. Furthermore, the filters “publication date: last five years” (applied at the time of the search to manage the expected high volume of retrieved studies given the broad scope of the search expression), “article type: meta-analysis”, “species: humans”, and “language: English” were used. The goal was to select the most relevant health effects associated with exposure changes in the scenarios to gather the measures of association needed for the quantitative RBA, i.e., to estimate the health impact of shifting to the alternative scenarios defined.

The flowchart (Figure S1, Online Resource) thoroughly depicts the methodology used to review and select the health effects to consider. The articles included were categorised into three groups according to the outcome: (i) *Cancer,* (ii) *Cardiovascular diseases*, (iii) *Diabetes Mellitus type 2 (T2D), metabolic, and weight-related outcomes*.

Along with the literature review, several steps were taken to narrow the studies obtained and select only the most robust associations to ensure a solid RBA. First, the data extraction was restricted to epidemiological studies with dose–response data (e.g. relative risks per x grams of food/nutrient) that reported significant associations for the incidence of diseases within the three outcome categories. Then, the selection of health outcomes was based on the strength and reliability of the evidence. The guidelines from the World Cancer Research Fund International (WCRF) [22] and the 2019 American College of Cardiology/American Heart Association (ACC/AHA) Guideline on the Primary Prevention of Cardiovascular Disease for Nutrition and Diet [23] for cancer and cardiovascular diseases, respectively, were referred to for this selection process, prioritising associations with evidence classified as high, strong, convincing, or probable. As no reference guideline document was found for T2D, metabolic and weight-related outcomes, a balance between the studies found with and without effect was first done to assess the inconsistency of available evidence between the different exposure and health effects. Then, umbrella reviews were considered [24, 25]. The final selection considered possible overlaps of exposure and outcomes to avoid overestimating the health impact (e.g., cardiovascular diseases comprise stroke, coronary heart disease and other outcomes; thus, in the selection of outcomes, only stroke and coronary heart disease were kept). Given that these consensus documents (i.e., WCRF report, ACC/AHA Guideline, and umbrella reviews) were used to assess the quality of evidence on the associations between exposures and health outcomes, the relative risks used in the RBA were those available in these studies.

Additionally, if plant-based analogues are classified as ultra-processed––Group 4 of the NOVA classification––and are used to replace mainly non-processed or minimally processed (NOVA Groups 1 and 3) foods, such as meat or fish, a noteworthy increase in UPF contribution (%) to total energy intake (TEI) is expected. Thus, systematic reviews were checked, and the most robust associations between health outcomes and UPF were extracted.

Table 2 presents the health outcomes selected, namely T2D, Coronary Heart Disease (CHD), Stroke, Colorectal cancer (CRC) and Overweight/obesity, and respective measures of association with the exposures varying within the scenarios.

**Table 2:** Health effects associated with the selected foods/nutrients and data inputs for the risk–benefit assessment: RR for the associations and DALYs for the health effects in the Portuguese population ≥ 20 years

Outcome category	Health outcome	Exposure^a^	Dose–response RR (95% CI)	References for RR	DALYs (95%CI)^b^
Cancer	Colorectal cancer	Red meat	1.12 (1.00; 1.25) per 100 g/day	[22]	92,516 (99,067; 85,412)
		Processed meat	1.16 (1.08; 1.26) per 50 g/day		
		Dairy	0.87 (0.83; 0.90) per 400 g/day		
		Fibre	0.93 (0.87; 1.00) per 10 g/day		
Cardiovascular diseases	CHD	Fibre	0.81 (0.73; 0.90) per 8 g/day	[54]	183,928 (197,702; 167,953)
		Fish	0.88 (0.79; 0.99) per 100 g/day	[55]	
		UPF	1.13 (1.02; 1.24) per 10 pp %TEI/day	[56]	
	Stroke	Fibre	0.90 (0.85; 0.95) per 8 g/day	[54]	227,739 (244,903; 206,535)
		Fish	0.86 (0.75; 0.99) per 100 g/day	[55]	
T2D, metabolic and weight-related outcomes	T2D	Red meat	1.17 (1.08; 1.26) per 100 g/day	[25]	153,229 (192,162; 119,969)
		Processed meat	1.37 (1.22; 1.54) per 50 g/day		
		Fibre	0.91 (0.87; 0.96) per 10 g/day		
		UPF	1.15 (1.06; 1.26) per 10 pp %TEI/day	[57]	
	Overweight/ obesity	UPF	1.13 (1.02; 1.24) per 10 pp %TEI/day		104089^c^ (147,873; 62,824)

CHD, coronary heart disease; T2D, diabetes mellitus type 2; UPF, ultra-processed foods; pp, percentual points; RR, relative risks; CI, confidence intervals; DALYs, Disability-Adjusted Life Years; %TEI, percentual contribution to the total energy intake

^a^Variability in the exposure to each parameter in each scenario is considered in the model as *Gamma* $\left({\alpha }_{i}, {\beta }_{i}\right)$ distributions (g/day)

^b^DALYs were extracted from the 2019 Global Burden of Disease (GBD) study

^c^DALYs for overweight/obesity in the GBD is represented by the risk factor “High Body-Mass Index” (DALYs_BMI_), which includes DALYs for multiple causes due to high BMI, including the other health outcomes considered in this RBA, namely T2D, CHD, stroke and colorectal cancer. To avoid double counting the burden of disease of these outcomes, the DALYs_BMI_ was corrected, and the DALYs for the causes T2D, CHD, stroke and colorectal cancer explained by the risk-factor factor “High Body-Mass Index” was subtracted from the DALYs_BMI_ for all causes

### Quantification of the health impact

To quantify the alternative scenarios’ health impact, considering the selected health outcomes, data on Disability-Adjusted Life Years (DALYs) for the population aged > 20 years old from Portugal from the 2019 Global Burden of Disease study (GBD) [26] was used, as this age range was the closest to the IAN-AF 2015–2016 adult age range cut-off. Table 2 displays the inputs from the GBD used in this study.

The health impact of each alternative scenario compared to the reference was estimated using a top-down approach. Accordingly, the RR and uncertainty intervals from the literature, presented in Table 2, were used to calculate the log-linear slope, β, and the RR for each scenario ($i$), according to the following equations:

$$\beta =\frac{{\text{ln}RR}_{literature}}{Dose}$$

$${RR}_{i}=\text{exp}\left(\beta \times {exposure}_{i}\right)$$

Log-linear associations between exposure and outcomes were assumed for the RR extracted from the literature, following the rationale from Berlin et al. [27]. Then, the Potential Impact Fraction (PIF) was calculated for each alternative scenario using the RR shift methodology [28].

$${PIF}_{alt}=\frac{{RR}_{alt}-{RR}_{ref}}{{RR}_{ref}}$$

The fraction of DALYs expected to change in each alternative scenario was estimated by multiplying the respective PIF by the sum of DALYs for all effects in the reference scenario obtained from the GBD 2019 study [26].

$$\Delta {DALY}_{alt}={PIF}_{alt}\times \sum_{HE}{DALY}_{ref}$$

Besides the overall ∆DALY estimate for each alternative scenario, the ∆DALYs stratified by sex were also computed. The inputs and results are presented as supplementary tables and figures,

### Sensitivity analysis—UPF

Given that there are conflicting arguments to consider PBAs as UPF or non-traditional UPF, two versions of $\Delta {DALY}_{alt}$ were estimated for the same alternative scenarios. One version includes the RR for associations between health outcomes and UPF, and another does not account for UPF variations.

### Quantification of the environmental impact

The SHARP Indicators Database (SHARP-ID) [29] was used to quantify the environmental impact in the reference and alternative scenarios in terms of greenhouse gas emissions (GHGE) and land use (LU) through a methodology described in a previous study [30]. Briefly, to merge SHARP-ID data with the IAN-AF 2015–2016 data, the FoodEx2 classification codes were used as the linking key. When a food item reported in IAN-AF 2015–2016 did not exist in SHARP-ID, an average value was assigned based on the closest hierarchical items. A precise match with the FoodEx2 code was achieved for 62% of the foods from IAN-AF 2015–2016 in the reference scenario. The indicator values were then multiplied by the amount of edible cooked food consumed per person, and the difference between each alternative and reference scenario ($\Delta {EnvironmentalImpact}_{alt}$) was calculated.

### Statistical analysis

In this probabilistic RBA, a two-dimensional Monte Carlo simulation (2nd-order MC) was used to deal with variability and uncertainty in estimating each alternative scenario’s health impact (∆DALY). Uncertainty parameters included the dose–response (RR), DALY values and the respective 95% confidence intervals (CI), described in the models as *Normal* and *Pert* distributions. Variability in the exposures for each scenario was described as *Gamma* distributions. The models included 1000 iterations to simulate variability and 5000 iterations to simulate uncertainty.

Moreover, the proportion of individuals with inadequate intake of some nutrients was estimated using the two-day average intake in each scenario. Inadequacies (%) were calculated for nutrients with a reference value defined by the European Food Safety Authority (EFSA), namely the *Average Requirement* (AR) and the *Adequate Intake* (AI) [31].

All analyses and models described were applied using R software version 3.6.2 [32].

## Results

### Comparison of alternative and reference scenarios: food and nutrient intake

**Fig. 1:**
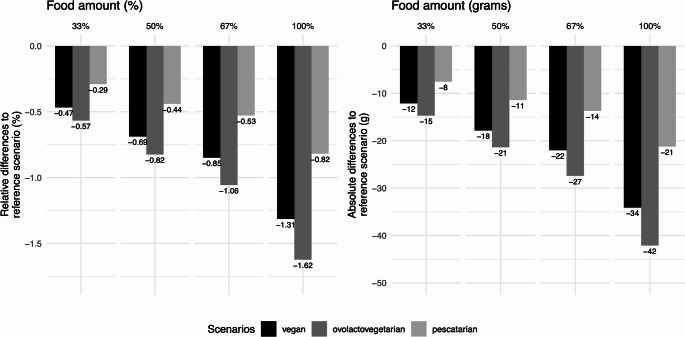
Differences (relative and absolute) in the amount (grams) of food consumed between the reference (i.e., the current consumption from the Portuguese Food, Nutrition and Physical Activity Survey, IAN-AF 2015–2016) and the isocaloric alternative scenarios. The differences were not statistically significant (*p* > 0.05)

The scenarios were designed to be isocaloric, meaning that the energy intake in the substitution scenarios was controlled to be equivalent to the reference scenario. However, the nutrient intake and the total amount of food consumed varied. As depicted in Fig. 1, the absolute and relative differences in the grams consumed between reference and alternative scenarios are negligible, with the most substantial discrepancy of 1.62%, equivalent to 42 g.

The differences between reference and alternative scenarios regarding nutrient intake and the prevalence of inadequacy are presented in Tables 3 and S2 (Online Resource) and Figures S3 and S4 from the Online Resource. A noticeable gradual increase in inadequate protein intake, vitamin B6, vitamin A, and riboflavin is observed when shifting from the reference to the alternative scenarios. The vegan scenario shows the highest prevalence of inadequate intakes of these nutrients. In the case of calcium, in the vegan scenario, as the proportion of items replaced by a PBA increases, there is a rise in the proportion of individuals with inadequate calcium intake (+ 12% to + 43%, for 33%–100%, respectively). In contrast, the inadequacy tends to decrease in the ovo-lacto-vegetarian or pescatarian scenarios. Iron and folate inadequacy reduces in all alternative scenarios. Regarding nutrients with AI as a reference value, Vitamin B12 and phosphorus intake decrease considerably in all alternative scenarios, particularly for a higher proportion of animal foods replaced by PBAs, increasing the likelihood of inadequacy. Conversely, dietary fibre and magnesium intake increase in all alternative scenarios, decreasing the likelihood of inadequacy.

**Table 3:** Relative differences in the prevalence of inadequate nutrient intake^a^ in each alternative scenario compared to the reference (consumption data from the Portuguese Food, Nutrition and Physical Activity Survey, IAN-AF 2015–2016)

Scenario level	Vegan				Ovolactovegetarian				Pescatarian			
% substitution	33	50	67	100	33	50	67	100	33	50	67	100
	%				%				%			
*Relative difference*^*b*^* in the prevalence of intake below the AR*												
Protein	**34.4**	**55.7**	**80.3**	**132.0**	**26.2**	**41.0**	**57.4**	**94.3**	**14.8**	**23.0**	**27.9**	**45.1**
Vitamin A	**22.4**	**34.7**	**47.6**	**73.5**	8.2	**13.2**	**14.5**	**24.6**	6.6	**11.0**	**12.0**	**20.2**
Vitamin C	− 1.8	− 2.7	-4.6	− 6.0	− 0.4	− 0.9	− 0.7	− 1.1	− 0.2	− 0.7	− 0.4	− 0.9
Vitamin B6	**38.0**	**60.9**	**80.2**	**137.0**	**37.5**	**53.6**	**71.4**	**126.0**	**31.3**	**45.8**	**57.3**	**101.0**
Riboflavin	**20.5**	**34.2**	**46.8**	**71.0**	8.4	**13.4**	**17.8**	**28.0**	8.2	**12.9**	**16.1**	**26.2**
Folates	**− 15.2**	**− 23.1**	**− 29.1**	**− 43.2**	− 9.9	**− 15.2**	**− 20.1**	**− 28.4**	− 9.4	**− 13.9**	**− 18.4**	**− 26.6**
Calcium	12.0	18.5	**26.3**	**43.0**	− 5.6	− 7.8	− 9.8	**− 14.6**	− 6.1	− 8.7	**− 10.5**	**− 15.9**
Iron	**− 35.6**	**− 50.0**	**− 61.0**	**− 73.7**	**− 22.0**	**− 33.1**	**− 34.7**	**− 50.8**	**− 16.9**	**− 26.3**	**− 27.1**	**− 39.0**
*Relative difference*^*b*^* in the prevalence of intake above the safe and adequate intake*												
Sodium	3.0	4.3	5.7	8.4	2.8	3.9	4.9	7.1	3.3	4.7	5.3	7.9
*Relative difference*^*b*^* in the prevalence of intake below the AI*												
Fibre	− 9.7	**− 15.3**	**− 21.9**	**− 33.6**	− 7.9	**− 12.4**	**− 17.3**	**− 26.9**	− 6.6	**− 10.7**	**− 14.1**	**− 22.1**
Vitamin B12	**23.9**	**37.5**	**49.4**	**72.4**	**20.3**	**32.2**	**43.9**	**66.0**	**13.5**	**20.7**	**26.1**	**40.4**
Vitamin D	1.3	1.6	2.0	2.9	1.2	1.7	2.2	2.9	0.1	0.2	0.1	0.3
Magnesium	**− 15.4**	**− 22.5**	**− 28.4**	**− 41.1**	**− 8.5**	**− 12.4**	**− 15.2**	**− 22.9**	**− 9.9**	**− 14.4**	**− 18.9**	**− 27.6**
Phosphorus	**52.0**	**96.0**	**140.0**	**292.0**	**28.0**	**44.0**	**56.0**	**108.0**	**20.0**	**24.0**	**24.0**	**52.0**
Potassium	4.4	6.6	7.5	**11.6**	2.9	4.1	6.7	**10.3**	1.2	1.8	2.5	4.4

AR, Average requirement; AI, Adequate intake; Ref, Reference scenario; Alt, Alternative scenario

^a^Prevalence of inadequate nutrient intake is defined as the proportion of individuals with a 2-day average intake below or above (in the case of sodium) the EFSA reference values

^b^Relative differences calculated as: $\left(\frac{Prevalence Ref}{Prevalence Alt}-1\right)\times 100$. Differences greater than ± 10% are highlighted in bold

### Health impact of alternative scenarios

**Fig. 2:**
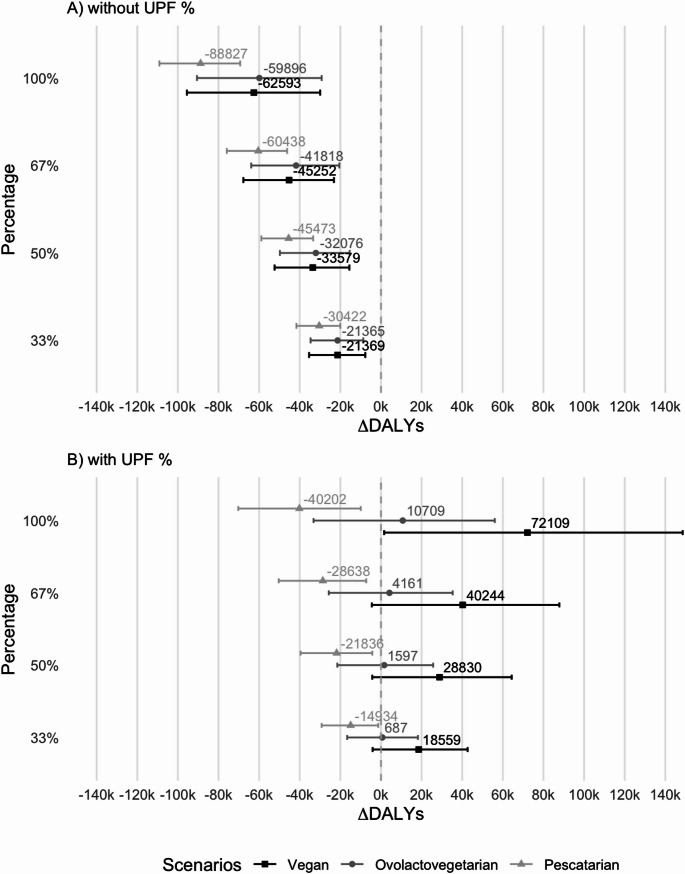
Health impacts (∆DALY) of alternative scenarios compared with the reference (i.e., the current consumption of the Portuguese population, according to the data from the National Food, Nutrition and Physical Activity Survey, IAN-AF 2015–2016). Figure 2A represents the results disregarding the possible effects of ultra-processed foods (UPF), while Fig. 2B represents the results including these effects

Regarding the overall health impact of the alternative scenarios considered, the results and their interpretation depend on including (or not) the epidemiological risk estimates associated with UPF consumption, except for the pescatarian scenario, as displayed in Fig. 2.

If PBAs are not considered UPF (Fig. 2A and Figure S2A), and thus the respective epidemiological risks are not included in the model, the transition to all alternative scenarios (vegan, ovolactovegetarian and pescatarian) would result in a health benefit for the Portuguese population (i.e., negative overall ∆DALY, i.e., considering all health outcomes), for both sexes. This benefit would increase as the proportion of items replaced also increases. The higher benefit would be obtained for a 100% pescatarian scenario (only meat substituted by PBA) with 88,827 DALYs (95%CI: 69,327; 109,090 DALYs per year) averted yearly.

The pescatarian was the only one that would also result in a health benefit if PBAs were classified as UPF and the risk estimates for the associated outcomes were considered (Fig. 2B). The higher benefit would once again be achieved for the 100% proportion of items replaced, but, in this case, the magnitude was smaller, and the uncertainty higher (∆DALY: -40,202 DALYs per year, 95%CI: − 70,268; − 9939 DALYs per year). However, as shown in Figure S2B, this is only the case for males. For females, no significant benefit is expected for this scenario. Transitioning to a vegan scenario in the Portuguese population resulted in health loss as the proportion of replaced items increased. If all (100%) animal-based foods were replaced by PBAs, 72,109 extra DALYs per year were estimated (∆DALY:72,109 DALYs per year, 95% CI: 1562; 148,533 DALYs per year). Nevertheless, the uncertainty around these figures was high, and the main health decay was estimated for females, with no significant results found for males (Figure S2B). The ovolactovegetarian scenario (PBAs considered UPF) did not result in a significant health impact for the Portuguese population.

### Environmental impact of alternative scenarios

**Fig. 3:**
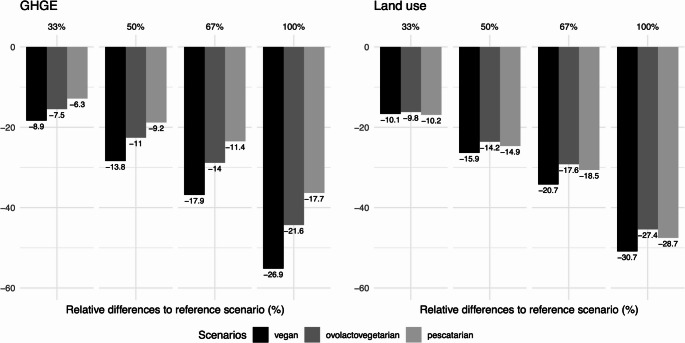
Environmental impacts (expressed as relative differences, %) of alternative scenarios compared with the reference (i.e., the current consumption of the Portuguese population, according to the data from the National Food, Nutrition and Physical Activity Survey, IAN-AF 2015–2016) for greenhouse gas emissions (GHGE) and land use (LU). All differences presented were statistically significant (*p* < 0.05)

The environmental impact of the transitions to the alternative scenarios expressed as relative differences (%) to the reference scenario for GHGE and LU is presented in Fig. 3. For GHGE, the transition to the vegan, ovo-lacto-vegetarian, and pescatarian scenarios would result in decreases ranging from 8.9% to 26.9%, 7.5% to 21.6%, and 6.3% to 17.7%, respectively (33% to 100% of items replaced). For LU, the transition to the vegan, ovo-lacto-vegetarian, and pescatarian scenarios would result in decreases ranging from 10.1% to 30.7%, 9.8% to 27.4%, and 10.2% to 28.7%, respectively (33%–100% of items replaced).

## Discussion

This study quantitatively compared the nutrient intake, health, and environmental impacts of hypothetical alternative dietary scenarios replacing animal foods with plant-based analogues, utilising the consumption data from the last Portuguese National Dietary Survey (2015–2016) as the baseline.

In the isoenergetic alternative scenarios, minimal discrepancies were observed in the total grams of food consumed within a day when PBAs substituted animal-based foods. This result hints at the scenarios’ practicality as possible real-life alternatives without disrupting the current dietary pattern, as described previously for PBAs [6, 7]. While the deviations in the quantity of food consumed were slight, the prevalence of inadequate protein, vitamins A, B6, riboflavin, and calcium intake (*calcium only for the vegan scenario*), and the probability of inadequacy for vitamins B12 and phosphorus increased compared to the reference, particularly in the *Vegan* scenario. These findings align with a previous study [33] and are expected as animal foods are important sources of these nutrients [12].

On the contrary, fibre, folate, magnesium, and iron intake increased in the alternative scenarios. If the fibre, folate, and magnesium results were expected, since plant-based foods typically contain high levels of these nutrients, the iron result was unexpected since iron inadequacy is described as a frequent drawback of vegetarian dietary patterns [12, 34]. This result was already described in a previous review of nutrient adequacy for plant-based diets [35]. It might emerge because most PBAs in our database are based on pulses and nuts and are probably iron-fortified. However, the potential lower bioavailability of iron in these products was not considered in the analyses, hampering a steady conclusion on this aspect, according to what has been suggested in a recent study [36].

Regarding DALYs, the health impact of the alternative scenarios depended on whether PBAs were assigned the epidemiological risk estimates associated with UPF consumption, that is, whether PBAs are considered UPF for the model. If PBAs were treated as UPF, the ovolactovegetarian and vegan scenarios would not result in net health benefits, with the vegan scenario even suggesting a potential public health risk at the highest replacement proportion studied, particularly among females. Notably, the classification of PBAs as UPF or non-UPF does not change their intrinsic nutritional composition or the actual effects on human health; rather, it affects the predicted health impact in the model by applying different epidemiological risk estimates. Arguments exist for both standpoints (i.e., considering PBAs as UPF or non-UPF). On the one hand, as mentioned in the introduction section, most epidemiological evidence linking UPF consumption to health outcomes primarily stems from studies involving foods with nutritional characteristics distinct from PBAs [15]. Accordingly, other authors disagree with classifying all PBAs as UPFs since it implies that all these products are nutritionally unbalanced, which has been evidenced otherwise [37].

On the other hand, apart from the nutritional profile, further mechanisms are proposed to explain the impact of UPF consumption on health outcomes that could be applied to PBAs. Notably, the so-called “matrix effect”, defined as the “food qualitative and holistic fraction”, is considered a central aspect influencing the health effects of foods. The importance of the matrix effect is underscored by the argument that two foods with the same composition but different matrices or structures may have diverse health effects (e.g., fruit vs. fruit juice) [38]. PBAs predominantly comprise a blend of unstructured, purified, fractionated, and highly processed ingredients, aligning with the classic attributes of UPF [39]. Several mechanisms are proposed to explain the role of the food matrix effect on health outcomes, namely its impact on organoleptic characteristics and palatability that could contribute to an accelerated eating rate and delayed satiety signalling, ultimately leading to increased overall food intake and other aspects such as impact on hormonal secretions, transit time, degree of chewing, and particle size after chewing. Furthermore, other considerations include the presence of contaminants introduced during processing and packaging (such as acrylamide, acrolein, BPA, and phthalates) [14, 38].

The NOVA classification, applied in this study to classify foods as UPF, is a practical, systematic approach to classify foods according to their nature, extent, and purposes of the submitted industrial processes. Current nutritional epidemiological research on the associations between the consumption of highly processed foods and health has mainly used the NOVA classification [40], and public health policymaking and guidelines are starting to cover food processing through this classification system [41, 42]. Nevertheless, there is criticism within the scientific community around the NOVA classification. Misclassification and inconsistencies have been described for NOVA, as well as considerable heterogeneity in the nutrient composition of foods from the same NOVA group [43]. In addition, the NOVA classification system is primarily descriptive, with a greater emphasis on sociocultural aspects than on the physical–chemical properties of foods. As a result, this focus may not fully capture the actual impact of food processing and the potential variations in the nutritional profiles of foods [44–46]. PBAs’ processing degree might even benefit PBAs over other non-processed plant-based protein sources. Unprocessed plant proteins may have lower nutritional quality than animal proteins due to the limited amounts of essential amino acids and lower digestibility [47]. It has been described, however, that food processing enhances the quality of plant-based proteins by degrading the cell wall, removing non-protein components, and inactivating or reducing antinutritional factors, such as protease inhibitors, phytates, and polyphenols [37].

These concerns around the NOVA functionality, reliability, and robustness build upon the uncertainties of considering the UPF approach in the current study. Moreover, assuming that the effect of all UPF on health outcomes is similar without considering possible differences in the diverse foods classified as UPF by NOVA may be a flawed approach. Still, until now, few longitudinal studies have focused on the specific effect of PBAs on health outcomes. Notwithstanding, a recent large multinational cohort study found an increased risk of multimorbidity of cancer and cardiometabolic diseases for total UPF consumption. Still, plant-based alternatives were not associated with risk in the subgroup analyses [48]. Accordingly, if PBAs yield different effects compared to conventional UPF, the findings of this study indicate that transitioning to any alternative dietary scenario (pescatarian, ovolactovegetarian, and vegan) would benefit the Portuguese population. The most considerable benefit would be observed quantitatively in the pescatarian scenario, where only meat is replaced by PBAs, with around 89 k DALYs saved (100% scenario), which could be explained in a simple manner that each year, almost 89 k individuals were expected to live one more healthy year if PBAs replaced meat. The comparatively lower quantitative benefit in the ovolactovegetarian and vegan scenarios suggests that the advantages of increasing fibre solely through plant-based substitutes of fish and dairy may not sufficiently counterbalance the simultaneous risks associated with reducing the original animal-based versions. These findings emphasise meat as the pivotal factor for the necessary dietary transition, particularly concerning the health impact, while minimising the required changes in the population’s dietary pattern and the potential inadequacy of some relevant nutrients like protein, vitamins A, B6, B12, riboflavin, and phosphorus. The results slightly differed by sex, with males, in general, benefiting further from the substitution of meat with a plant-based counterpart. This result may be explained by the current dietary pattern differences between males and females, as males generally have a higher animal-based food consumption than females, as shown in previous studies from this population [49]. Also, the incidence of the health outcomes considered, along with the respective burden of disease, estimated in DALYs, differs between sexes [26] and helps explain the differences found.

Regarding the environmental impact, both indicators observe the most substantial changes in the vegan scenario. However, upon comparing the vegan and pescatarian scenarios, the magnitude of difference may not justify a complete transition, given that the substitution of meat alone already leads to a significant reduction in both GHGE and LU. There are still some considerations regarding this assessment. Namely, the information on the impacts of PBAs is limited in the dataset used (SHARP-ID). Consequently, the *Tofu* and *Textured-Soy protein* values from the SHARP-ID were attributed to all PBAs. A recent study that reviewed the environmental impact of meat substitutes has reported varying environmental impacts in plant-based meat products depending on product formulation, especially for egg-containing PBAs [8]. However, the effect of this limitation in the results most likely does not affect our conclusions, as they are aligned with the ones from the same study when comparing the environmental impact of PBAs with meat.

To our knowledge, this is the first quantitative risk–benefit assessment evaluating the impact of substituting animal-based foods with plant-based analogues using a health composite metric, the DALYs. By minimising the disruption of the current dietary patterns, the transition from animal-based foods to PBAs reflects a possible, feasible and more realistic approach for the first stage of the necessary dietary transition to a more sustainable dietary pattern. This assessment used a second-order Monte Carlo simulation to address both exposure variability and uncertainty in dose–response and DALYs data. However, additional sources of uncertainty could change the quantitative health impact estimates (∆DALY) and, thus, should be discussed. Namely, there are other constituents of PBAs, other than fibre, that can impact health outcomes and are not being considered, such as sodium. Many PBAs have been reported to contain substantial sodium levels [50, 51], which can introduce potential unquantified health risks of these foods, as sodium is associated with increased risk of cardiovascular diseases [52]. Quantifying sodium using food consumption data is challenging mainly due to the variability in discretionary salt use in recipes and at the table during meals [53]. In the food consumption data from IAN-AF 2015–2016, an average amount of added salt is considered in meat, fish, and egg recipes to avoid underestimating sodium intake. The estimated increase in sodium intake in the alternative scenarios was not very pronounced, as depicted in Figure S4 (Online Resource) and indirectly shown in Tables 3 and S2 (Online Resource). Still, it remains unclear whether substituting these animal-based foods (typically with low sodium levels) with the respective PBA would decrease discretionary salt use, further smoothing the slight differences in sodium intake. For these reasons, it was decided not to include sodium in the quantitative RBA.

Furthermore, the results should be carefully interpreted, as other health effects could have been missed in the quantitative assessment by changing exposures in the scenarios. Still, the selection of health outcomes was based on a thorough methodology, selecting only the effects where high-quality dose–response data were available. The degree of evidence was agreed upon by international agencies or umbrella reviews [22–25] as high, strong, convincing, or probable, strengthening the confidence that these results reflect the best available evidence thus far. Furthermore, nutrient adequacy was assessed to complement the quantitative ∆DALY results and give further insight into the scenarios’ diet quality by considering additional possible trade-offs. Also, the results are highly dependent on the definition of alternative scenarios. In this study, three alternative scenarios (*Vegan*, *Ovolactovegetarian,* and *Pescatarian*) with different proportions of substitution (proportions: 33%, 50%, 67%, 100%) were considered. Although the definition of these proportions was ad hoc, they allowed us to capture a wide range of substitution levels, providing a representative gradient of substitution effects. Still, other cut-offs (e.g., 25%, 50%, 75%, 100%) could have been used as well, and would yield slightly different results. Nevertheless, we do not expect such a change to affect the overall interpretation and conclusions of the study materially.

Moreover, this study focused specifically on Portugal, its general population’s current consumption of animal-based foods [16, 17], and used country-specific data on DALYs for the selected outcomes [26]. Thus, the estimated health and environmental impact of these scenarios may differ in different settings, with varying consumption patterns of animal-based foods and other disease burden values concerning the selected health outcomes, as described in a previous modelling study [2].

An additional remark must be made regarding the terminology used to define the scenario levels in this study, namely the definitions of “vegan”, “ovo-lacto-vegetarian”, and “pescatarian”. These terms were used to facilitate the description of results and the interpretation of the view of the foods replaced by PBAs in each alternative scenario. Still, they should not be regarded as a faithful representation of the traditional definition of these dietary patterns. These dietary patterns are frequently described as nutritionally balanced, in line with available evidence on healthy eating [2]. This may not necessarily be true for the alternative scenarios modelled in the current study.

In conclusion, this study’s findings support PBAs as a viable alternative to meat products, resulting in public health benefits for the Portuguese population and reduced environmental impact if PBAs replace at least one-third of meat products. This substitution would significantly decrease the Portuguese population’s DALYs, regardless of whether the model included the epidemiological risk estimates associated with UPF consumption to PBAs, and would result in the lowest decrease in protein intake of all scenarios tested. Despite the clear environmental benefits, the health impact of substituting other animal-based foods, such as fish or dairy, with PBAs is still unclear. Future epidemiological research, specifically focusing on the consumption of PBAs, would be essential to clarify the effects of these foods on health outcomes.

## Electronic supplementary material

Below is the link to the electronic supplementary material.

Supplementary Material 1
