# Supplementary material for: Health and environmental impacts of shifting to plant-based analogues: a risk-benefit assessment
Source: Eur J Nutr. 2025 Jul 5;64(5):234. doi: 10.1007/s00394-025-03749-z (PMC12228673; doi:10.1007/s00394-025-03749-z)
Supplement: Supplementary file 1 — Supplementary Material 1 [file 394_2025_3749_MOESM1_ESM.pdf]

**Supplementary Material**
European Journal of Nutrition

# Health and Environmental Impacts of Shifting to Plant-Based Analogues: A Risk-Benefit Assessment

Catarina Carvalho[1,2,3], Daniela Correia[1,2,4], Sofia Almeida Costa[1,2], Rita Pereira[1,2], Andreia Oliveira[1,2,4], Elisabete Pinto[1,5], Carla Lopes[1,2,4], Duarte Torres[1,2,3]

[1] EPIUnit – Instituto de Saúde Pública, Universidade do Porto, Porto, Portugal.
[2] Laboratório para a Investigação Integrativa e Translacional em Saúde Populacional (ITR), Porto, Portugal
[3] Faculdade de Ciências da Nutrição e Alimentação, Universidade do Porto, Porto, Portugal.
[4] Departamento de Ciências da Saúde Pública e Forenses e Educação Médica, Faculdade de Medicina, Universidade do Porto, Porto, Portugal.
[5] CBQF-Centro de Biotecnologia e Química Fina-Laboratório Associado, Escola Superior de Biotecnologia, Universidade Católica Portuguesa, Porto, Portugal.

Corresponding author:
Catarina Carvalho
Email: catarina.carvalho@ispup.up.pt
ORCID ID: 0000-0002-1790-7421
Address: EPIUnit – Instituto de Saúde Pública da Universidade do Porto, Rua das Taipas, nº 135, 4050-600 Porto, Portugal. T: +351 222061820

**Table of contents**

**Figure S1.** Flowchart of the selection of articles of associations between relevant exposures and outcomes. ........ 3

**Figure S2.** Health impacts (ΔDALY) of alternative scenarios compared with the reference, per sex. Figure 2A represents the results disregarding the possible effects of ultra-processed foods (UPF) for females (A1) and males (A2), while Figure 2B represents the results including the UPF effects, for females (B1) and males (B2). ........ 4

**Figure S3.** Differences (%) in energy from ultra-processed food (UPF) and macronutrient intake between the reference (current consumption from Portuguese Food and Physical Activity Survey) and alternative scenarios of Plant-Based Analogues consumption. ........ 5

**Figure S4.** Differences (%) in micronutrient intake between the reference (current consumption from Portuguese Food and Physical Activity Survey) and alternative scenarios of Plant-Based Analogues consumption ........ 6

**Table S1.** Disability-Adjusted Life Years (DALYs) for the Portuguese population aged over 20 years for the outcomes under study, stratified by sex and extracted from the Global Burden of Disease study. ........ 7

**Table S2.** Prevalence of nutrient intake below the reference values in each scenario, in the Portuguese population, using data from the Portuguese Food and Physical Activity Survey (IAN-AF 2015-2016). ........ 8

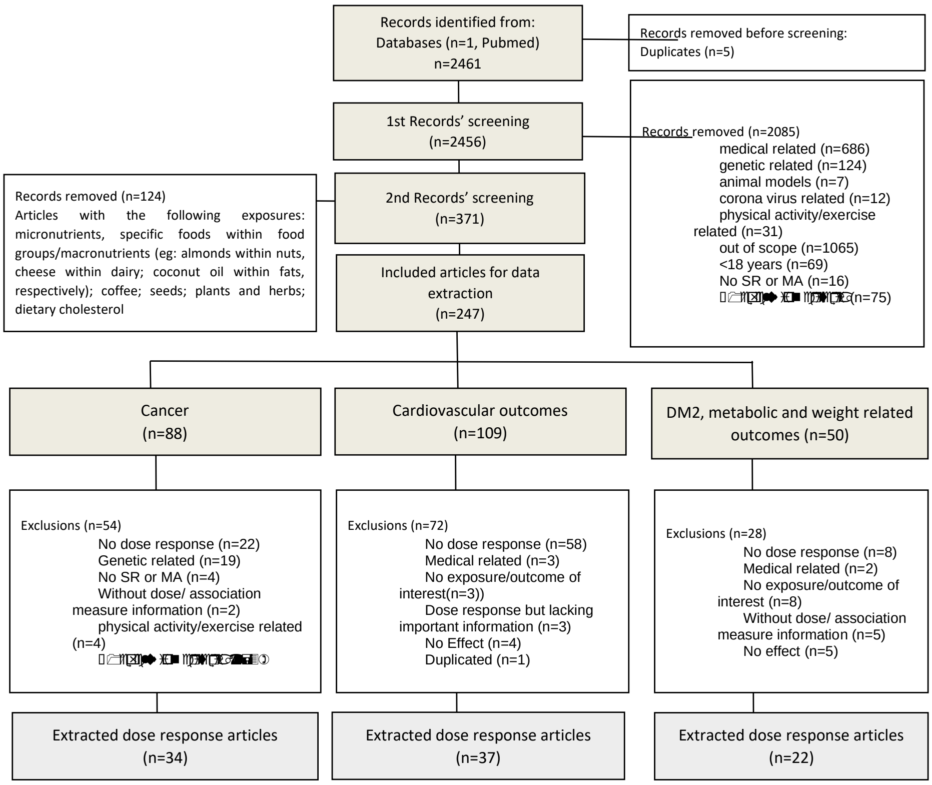


**Figure S1.** Flowchart of the selection of articles of associations between relevant exposures and outcomes.

The final search expression was: *(((“food”[tw], "animal-based”[tw] OR "meat”[tw] OR “fish”[tw] OR “eggs”[tw] OR "dairy products"[tw] OR “milk”[tw] OR “milk subsBtutes”[tw] ) OR (“plant-based”[tw] OR “legume*”[tw] OR “pulses”[tw] OR “nuts”[tw] OR “grain”[tw] OR “whole grains”[tw] OR “meat alternaBves”[tw] OR “plant-based meat alternaBves”[tw]) OR ("dietary proteins"[tw] OR “dietary fats”[tw] OR "faFy acids"[tw] OR “saturated fat”[tw] OR “FaFy Acids, Omega-3 +”[tw] OR “FaFy Acids, Omega-6 +”[tw] OR “omega-3”[tw] OR “cholesterol”[tw] OR “fiber”[tw] OR “dietary fiber”[tw] OR “micronutrients”[tw] OR “iron”[tw] OR “zinc”[tw] OR “vitamin B 12”[tw] OR “calcium”[tw] OR “sodium”[tw] OR "sodium, dietary"[tw])) AND (“neoplasms"[tw] OR “cancer”[tw] OR "cardiovascular diseases"[tw] OR “obesity”[tw] OR “mortality”[tw] OR “diabetes mellitus”[tw] OR “health outcomes”[tw] OR disease[tw] OR “disorders”[tw]))*

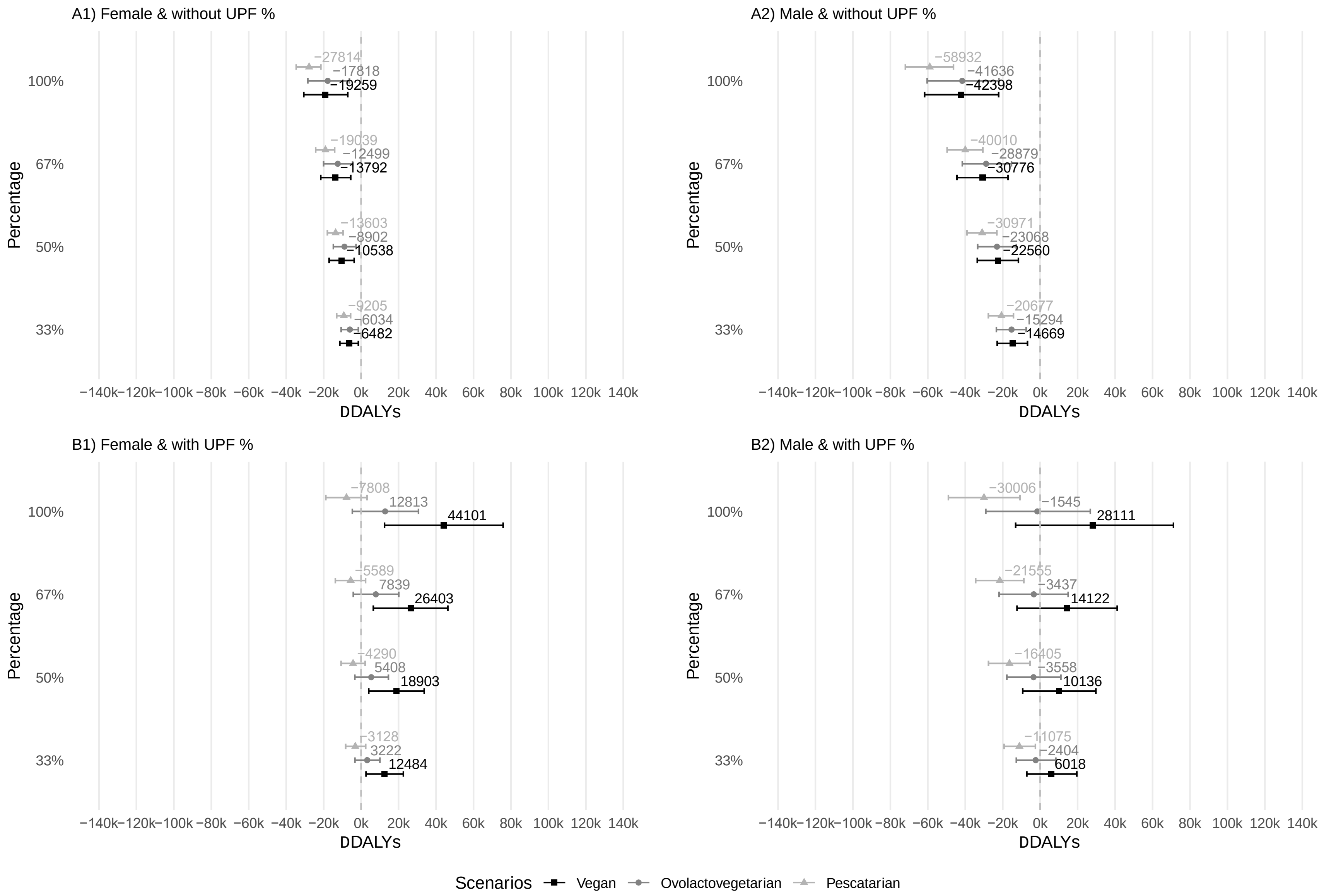


**Figure S2.** Health impacts (∆DALY) of alternative scenarios compared with the reference, per sex. Figure 2A represents the results disregarding the possible effects of ultra-processed foods (UPF) for females (A1) and males (A2), while Figure 2B represents the results including the UPF effects, for females (B1) and males (B2).

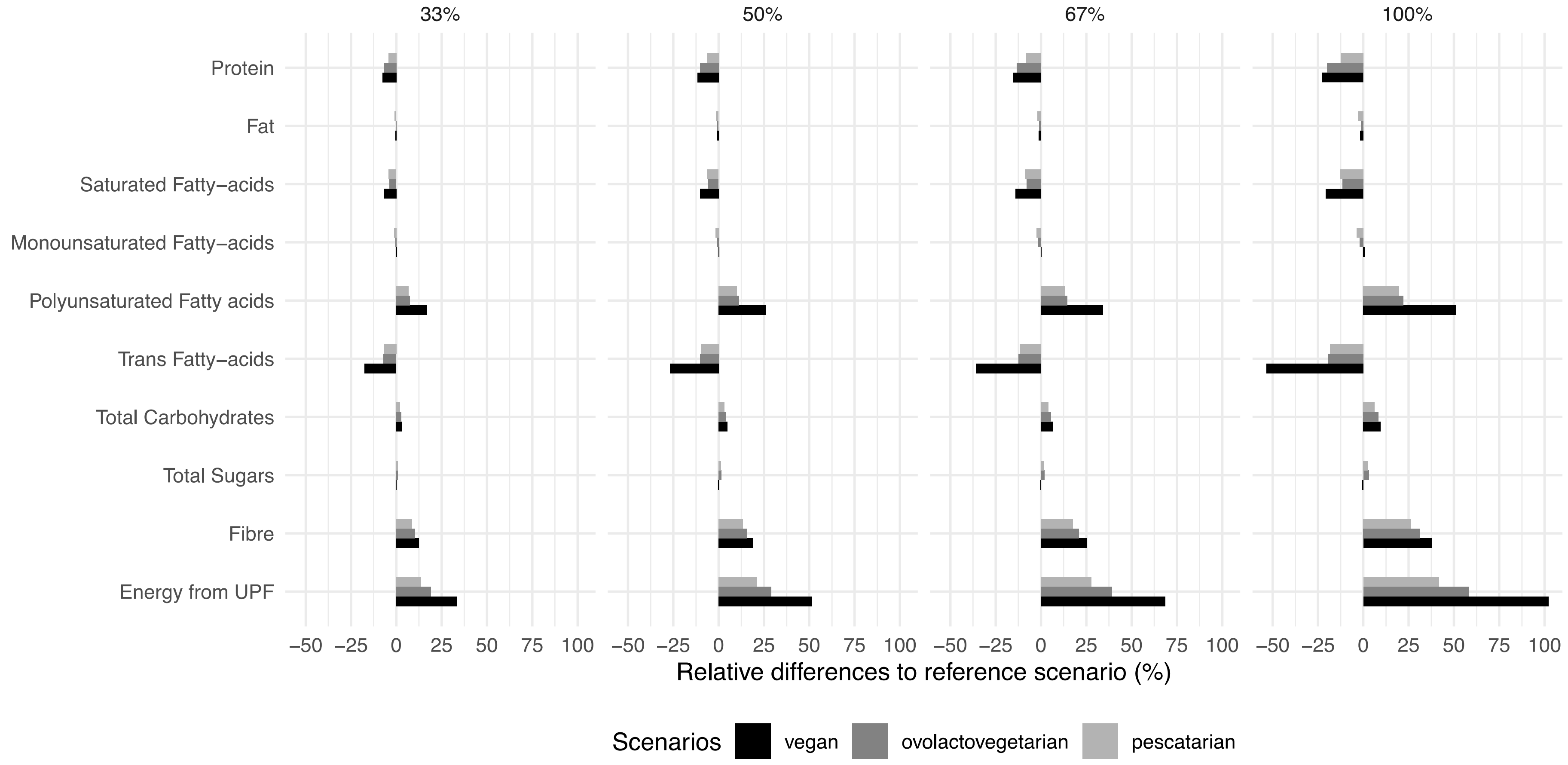


**Figure S3.** Differences (%) in energy from ultra-processed food (UPF) and macronutrient intake between the reference (current consumption from Portuguese Food and Physical Activity Survey) and alternative scenarios of Plant-Based Analogues consumption.

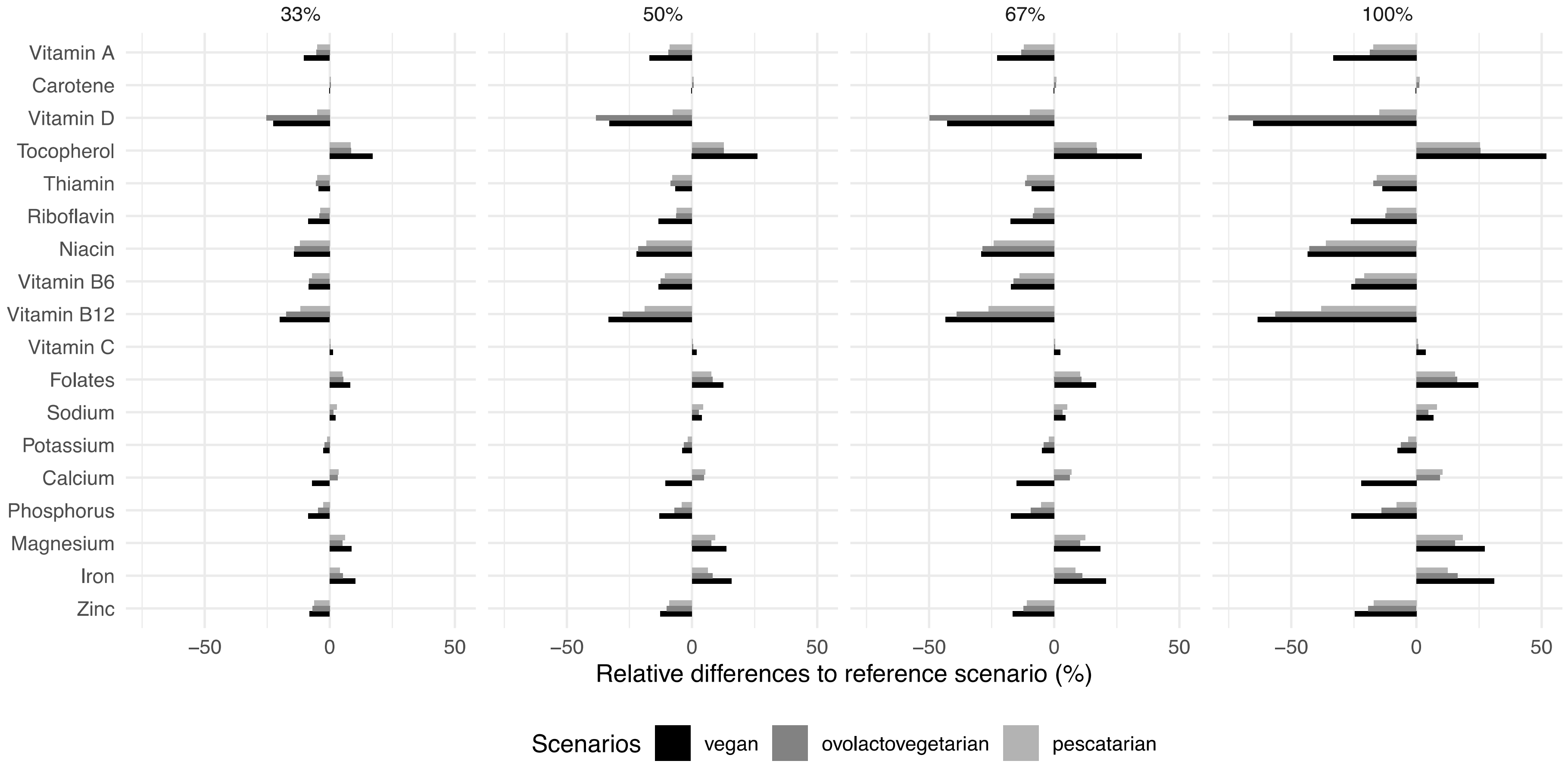


**Figure S4.** Differences (%) in micronutrient intake between the reference (current consumption from Portuguese Food and Physical Activity Survey) and alternative scenarios of Plant-Based Analogues consumption.

**Table S1.** Disability-Adjusted Life Years (DALYs) for the Portuguese population aged over 20 years for the outcomes under study, stratified by sex and extracted from the Global Burden of Disease study.

| Outcome category | Health outcome | DALY (95%CI)[2] | |
|---|---|---|---|
| | | **Females** | **Males** |
| Cancer | Colorectal cancer | 36105 (30754; 41473) | 53900 (47684; 61298) |
| Cardiovascular diseases | CHD | 67712 (56962; 73798) | 110168 (103925; 116462) |
| | Stroke | 107113 (90355; 117239) | 99216 (91723; 104983) |
| DM2, metabolic and weight-related outcomes | DM2 | 76726 (61167; 96878) | 70842 (56561; 88105) |
| | Overweight/ obesity | 83934 (24712; 141387) [3] | 49234 (18556; 80967) [3] |

Abbreviations: CHD – coronary heart disease; DM2 – diabetes mellitus type 2; UPF – ultra-processed foods; pp – percentual points; RR: relative risks; CI: confidence intervals; DALY: Disability-Adjusted Life Years; %TEI – percentual contribution to the total energy intake;

[1] Variability in the exposure to each parameter in each scenario is considered in the model as *Gamma* $(\alpha_i, \beta_i)$ distributions (g/day).
[2] DALY values were extracted from the 2019 Global Burden of Disease study (GBD)
[3] DALY for overweight/obesity in the GBD is represented by the risk factor “High Body-Mass Index” ($DALY_{BMI}$), which includes DALY for multiple causes due to high BMI, including the other health outcomes considered in this RBA, namely DM2, CHD, stroke and colorectal cancer. To avoid double counting the burden of disease of these outcomes, the $DALY_{BMI}$ was corrected, and the DALY for the causes DM2, CHD, stroke and colorectal cancer explained by the risk-factor factor “High Body-Mass Index” was subtracted from the $DALY_{BMI}$ for all causes.

**Table S2.** Prevalence of nutrient intake below the reference values in each scenario, in the Portuguese population, using data from the Portuguese Food and Physical Activity Survey (IAN-AF 2015-2016).

| | *Ref* | *Vegan* | | | | *Ovolactovegetarian* | | | | *Pescatarian* | | | |
|---|---|---|---|---|---|---|---|---|---|---|---|---|---|
| | | *33* | *50* | *67* | *100* | *33* | *50* | *67* | *100* | *33* | *50* | *67* | *100* |
| | | % | | | | % | | | | % | | | |
| *Prevalence (%) of intake below the AR* | | | | | | | | | | | | | |
| Protein | 12.2 | 16.4 | 19.0 | 22.0 | 28.3 | 15.4 | 17.2 | 19.2 | 23.7 | 14.0 | 15.0 | 15.6 | 17.7 |
| Vitamin A | 31.7 | 38.8 | 42.7 | 46.8 | 55.0 | 34.3 | 35.9 | 36.3 | 39.5 | 33.8 | 35.2 | 35.5 | 38.1 |
| Vitamin C | 45.2 | 44.4 | 44.0 | 43.1 | 42.5 | 45.0 | 44.8 | 44.9 | 44.7 | 45.1 | 44.9 | 45.0 | 44.8 |
| Vitamin B6 | 19.2 | 26.5 | 30.9 | 34.6 | 45.5 | 26.4 | 29.5 | 32.9 | 43.4 | 25.2 | 28.0 | 30.2 | 38.6 |
| Riboflavin | 40.4 | 48.7 | 54.2 | 59.3 | 69.1 | 43.8 | 45.8 | 47.6 | 51.7 | 43.7 | 45.6 | 46.9 | 51.0 |
| Folates | 55.3 | 46.9 | 42.5 | 39.2 | 31.4 | 49.8 | 46.9 | 44.2 | 39.6 | 50.1 | 47.6 | 45.1 | 40.6 |
| Calcium | 57.4 | 64.3 | 68.0 | 72.5 | 82.1 | 54.2 | 52.9 | 51.8 | 49.0 | 53.9 | 52.4 | 51.4 | 48.3 |
| Iron | 11.8 | 7.6 | 5.9 | 4.6 | 3.1 | 9.2 | 7.9 | 7.7 | 5.8 | 9.8 | 8.7 | 8.6 | 7.2 |
| *Prevalence (%) of intake above the safe and adequate intake* | | | | | | | | | | | | | |
| Sodium | 78.9 | 81.3 | 82.3 | 83.4 | 85.5 | 81.1 | 82.0 | 82.8 | 84.5 | 81.5 | 82.6 | 83.1 | 85.1 |
| *Prevalence (%) of intake below AI* | | | | | | | | | | | | | |
| Fibre | 84.9 | 76.7 | 71.9 | 66.3 | 56.4 | 78.2 | 74.4 | 70.2 | 62.1 | 79.3 | 75.8 | 72.9 | 66.1 |
| Vitamin B12 | 54.7 | 67.8 | 75.2 | 81.7 | 94.3 | 65.8 | 72.3 | 78.7 | 90.8 | 62.1 | 66.0 | 69.0 | 76.8 |
| Vitamin D | 97.2 | 98.5 | 98.8 | 99.1 | 100.0 | 98.4 | 98.9 | 99.3 | 100.0 | 97.3 | 97.4 | 97.3 | 97.5 |
| Magnesium | 71.5 | 60.5 | 55.4 | 51.2 | 42.1 | 65.4 | 62.6 | 60.6 | 55.1 | 64.4 | 61.2 | 58.0 | 51.8 |
| Phosphorus | 2.5 | 3.8 | 4.9 | 6.0 | 9.8 | 3.2 | 3.6 | 3.9 | 5.2 | 3.0 | 3.1 | 3.1 | 3.8 |
| Potassium | 65.3 | 68.2 | 69.6 | 70.2 | 72.9 | 67.2 | 68.0 | 69.7 | 72.0 | 66.1 | 66.5 | 66.9 | 68.2 |

Abbreviations: AR – Average requirement; AI – Adequate intake; Ref – Reference scenario
